# The validation of organisational culture assessment instrument in healthcare setting: results from a cross-sectional study in Vietnam

**DOI:** 10.1186/s12889-020-8372-y

**Published:** 2020-03-12

**Authors:** Nguyen Van Huy, Nguyen Thi Hoai Thu, Nguyen Le Tuan Anh, Nguyen Thanh Hai Au, Nguyen The Phuong, Nguyen Thi Cham, Pham Duc Minh

**Affiliations:** 1 Graduate School of Public Health, St. Luke’s International University, Tokyo, Japan; 2 Institute for Preventive Medicine and Public Health, Hanoi Medical University, Hanoi, Vietnam; 3 Department of Population and Quantitative Health Sciences, University of Massachusetts Medical School, Massachusetts, USA; 4 Department of Health Management and Organization, Institute for Preventive Medicine and Public Health, Hanoi Medical University, Hanoi, Vietnam; 5 Quang Nam General Central Hospital, Tam Hiep Commune, Nui Thanh District, Quang Nam Province, Vietnam; 6 Preventive Medicine, Public Health Student at Graduate School of Public Health, St. Luke’s International University, Tokyo, Japan; 7 Training Center and Scientific Management, National Institute of Occupational Health and Environment, Hanoi, Vietnam; 8 Burnet Institute, 85 Commercial Road, Melbourne, Australia; 9 Department of Epidemiology and Preventive Medicine, School of Public Health and Preventive Medicine, Faculty of Medicine, Nursing and Health Sciences, Monash University, Melbourne, Australia

**Keywords:** Organisational culture (OC), Organisational culture assessment instrument (OCAI), Healthcare setting, Developing countries, Vietnam

## Abstract

**Background:**

Organisational culture (OC) has increasingly become a crucial factor in defining healthcare practice and management. However, there has been little research validating and adapting OCAI (organisational culture assessment instrument) to assess OC in healthcare settings in developing countries, including Vietnam. The purpose of this study is to validate the OCAI in a hospital setting using key psychometric tests and confirmatory factor analysis (CFA).

**Methods:**

This is a cross-sectional study. Self-administered structured questionnaire was completed by 566 health professionals from a Vietnamese national general hospital, the General Hospital of Quang Nam province. The psychometric tests and CFA were utilized to detect internal reliability and construct validity of the instrument.

**Results:**

The Cronbach’s alpha coefficients (α-reliability statistic) ranged from 0.6 to 0.8. In current culture, the coefficient was 0.80 for clan and 0.60 for adhocracy, hierarchy and market dimension, while in expected culture, the coefficient for clan, adhocracy, hierarchy, and market dimension was 0.70, 0.70, 0.70 and 0.60, respectively. The CFA indicated that most factor loading coefficients were of moderate values ranging from 0.30 to 0.60 in both current and expected culture model. These models are of marginal good fit.

**Conclusions:**

The study findings suggest that the OCAI be of fairly good reliability and construct validity in measuring four types of organisational culture in healthcare setting in resource-constrained countries such as Vietnam. This result is a first step towards developing a valid Vietnamese version of the OCAI which can also provide a strong case for future research in the field of measuring and managing organisational culture.

## Background

The relationship between OC (organisational culture) and performance of an organisation has been an area of a growing research interest. We selected the theory framework of Competing Values Framework (CVF), which was proposed by Quinn and Rohrbaugh [19]. This theory has furthered the measurement and comprehension of OC structure. The CVF is divided in two dimensions, forming four major clusters (clan, adhocracy, market, and hierarchy) [3]. The first dimension distinguishes the dynamism, discretion and flexibility from control, order and stability. The second one discriminates the unity, integration and internal orientation from rivalry, differentiation and external orientation. The competing values in each quadrant are the reason for this framework’s name: the Competing Values Framework. We chose this theoretical model for a variety of reasons. First, it is an evidence-based framework as it was developed based on research showing both face and empirical validity. Second, the CVF can fit diverse types of organisational settings and is utilised to measure types, congruence, and strengths of OC using commonly associated terms: the core cultural values, interpretations and assumptions that characterise organisations [3]. It also provides a framework for studying and understanding OC that can reflect a mixture of multiple cultural types as well as diverse characteristics of a particular cultural type [6, 8, 13]. Last, but not the least, as OC tends to develop over time with the adaption and responses of members to the environment, the CVF is a conceptual foundation that can fit a variable context and as a result be applied for research and facilitation of OC change and OCAI being discussed below is among the tools developed from such a framework [19].

Clan Culture (CC), which is identified by the flexibility and internal focus aspects of the CVF, is typical of a family-style organisation with a friendly working environment. Leaders play the role of mentors and facilitators. Employees are committed, and focus on the long-term benefit of individual development. Teamwork, cohesion, and loyalty are important aspects of this culture. Adhocracy Culture (AC), characterised by the flexibility and external focus aspects of the CVF, is typical of a dynamic and creative working environment. Leaders are seen as innovators and risk takers. Employees accept the challenge, want to make a difference and can be seen as very aggressive, with a desire to lead. Commitment to experimentation and innovations, high specialisation and rapid change of organisation are the key aspects of the Adhocracy Culture. The internal focus and stability aspects of the CVF are describe in Hierarchy Culture (HC), which is a serious and organised work environment, similar to governmental organisations. Leaders are proud of their workplace and play the role of coordinators, supervisors. Employees are highly aware and compliant to the principles and procedures of the organisation. Stable development, efficiency and control, rules and policies are the key aspects of Hierarchy culture. The last one - Market Culture (MC), which is defined by the stability aspects and external focus of the CVF, is typical of a results-oriented workplace. Managers are hard-driving competitors and producers. Employees focus on success and achievement. The important aspects of this culture are long-term concern for competitiveness and winning [3].

To measure the current OC and its cultural preferences, Cameron and Quinn have developed the Organizational Culture Assessment Instruments (OCAI) based on the CVF. This instrument was created in order to measure the OC aspects in the present situation and to meet the wishes of employees [3]. OCAI, a classification approach [12] was developed to evaluate OC with six core attributes: Dominant Characteristics; Organisational Leadership; Management of Employee; Organisation Glue; Strategic Emphases; Criteria of Success. The questionnaire includes 24 items divided into four alternatives, which correspond with the four cultural types labelled Clan, Adhocracy, Market, and Hierarchy [3].

Many studies still have limited evidence for validating an instrument measuring psychology properties such as OCAI [5]. Previous studies used Vietnamese version of OCAI to measure OC in Vietnam. However, it requires more effort than a literal translation to develop an acceptable instrument for another cultural group [11, 23]. Most of these studies did not clarify the way of each item in the OCAI was translated and the translated version was validated by which methods. When the OCAI is used in a different country, culture and sample from the original instrument, its psychometric properties, including reliability and validity, have to be re-examined [7]. Unfitting translation processes could lead to biased or misguided study outcomes [4, 15]. In addition, there has been a growing need for standard and validated practices for a translated psychometric scale in a healthcare setting in developing countries such as Vietnam. Therefore, we conducted this study in order to validate the OCAI in a healthcare setting as a first step towards establishing a valid a Vietnamese version of the OCAI. This study also serves as a valid basis for future studies in the field of measuring and managing OC.

## Methods

### Study design

This cross-sectional design study employed quantitative research methods and was conducted in Quang Nam province’s General hospital from April 2016 to July 2017.

### Participants

All health staffs working in the hospital were approached for data collection. Of 701 staff eligible for interview, 566 people agreed to participate in the research, reaching a participation rate of 80.7%. The sample of 566 participants met the minimum power requirement for analysis.

### Research instrument

The OCAI was chosen in this study [3] because it’s abilities of describing the culture depending on alignments and identifying the expected pattern between culture factors and other organizational variables of interest. In addition, OCAI is one of the instruments can assess culture with demonstrated adequate internal consistencies and evidences for aggregating individual data to be representative of the organisation as a whole [10].

The OCAI developed by Cameron and Quinn [3] was translated and reworded to create an appropriate and comprehensive Vietnamese version. We employed self-administered questionnaire to collect data. Eligible participants were invited to the hospital’s meeting room of and were introduced to the purpose of this study as well as the instrument. Researchers ensured the privacy of the meeting place and no exchange of information among participants was allowed during the survey. All questionnaires were completed by participants and transferred to research team.

### Key measures and data analysis

The six attributes of OC, Dominant Characteristics, Organisational Leadership, Management of Employee, Organisation Glue, Strategic Emphases, Criteria of Success, were evaluated by using OCAI with 24 items [3]. Within each component, there were 4 statements, each of which represents one type of OC. Participants were asked to distribute 100 points between 4 statements to indicate their organisational relevance. First they were asked about culture perceptions of their current organisations by 24 item questions/statements. Respondents were then asked again but for their ideal or expected organisational practices. After all steps, 24 items were regrouped to form 4 types of OC, CC, AC, HC and MC, each of which had 6 statements. Because of its compromise, a higher score of one type of OC means a lower score of another one.

The instrument was assessed according to two main criteria, reliability and validity. The correlations between the four dimensions of current and expected organisational culture were tested to confirm the consistency of the CVF and that the data was appropriate for factor analysis. Cronbach’s alpha coefficients (α reliability statistic) were calculated for all the culture types, and the internal consistency indices were demonstrated as sufficient as they ranged from 0.6 to 0.8 [3]. Two kinds of validity were analysed in our study, construct and face validity. Face validity was satisfied based on the results of two activities, a pilot survey among 30 participants and a review by senior researchers involved in this study. There was no much change in instrument contents after pilot. To assess construct validity, CFA (confirmatory factor analysis) was utilized for both the current and ideal organizational culture data. CFA was performed not only to assess construct validity, but also to refine measurement instruments. Construct validity refers to the extent to which an instrument measures what it means to measure as defined by a theory [18]. The CFA was conducted two times using data of the current and expected OC to evaluate whether the culture conformed to the hypothesised OCAI model. The unidimensional models with all indicators loading onto a single factor then were used to assess the fit of the four-factor model. Our data is suitable for factor analysis as we achieved a more-than-23-time ratio of our sample of 566 participants over 24 OCAI items (the minimum required of at least 5 to 10 times the amount of items in the CFA model [14], most of the correlations were of statistically significant moderate levels and there were no outliers in the data [22].

Data was checked and entered by Epi data 3.1 and was analysed by using STATA 10.0. Prior to the analysis, all assumptions were tested to ensure the model fit by considering the value of χ2/df, Standardized Root Mean Square Residual (SRMR), Comparative fit index (CFI) and Root mean square error of approximation (RMSEA). AMOS version 20.0 was used to test model re-assessment for both current and expected cultural model that created the factor loading coefficients between each item and between dimensions.

### Research ethics

This study was ethically and scientifically reviewed and approved by Hanoi Medical University according to Decision No. 5403/QD-DHYHN dated 06/12/2016. All participants received information of the purpose and methods and their right to refuse participation at any time. We obtained their verbal informed consent as the principal investigator is currently working at this hospital and all participants agreed to support the study and as this study is part of research agenda of the hospital to improve healthcare quality. They were re-affirmed that their participation was voluntary, that their anonymity was maintained, and that their refusal would not affect their health care.

## Results

### The selected socio-demographic characteristics of the sample

Among the 566 respondents enrolled in the survey, majority are female (72.4%), quite young (mean age of 31.9 years old) and married (80.7%). Most of respondents have intermediate degree (39.4%), followed by college degree (33.6%) and university degree (27%). The ratio of managers and staff is one-ninth (10.3 and 90.7%) and most respondents having more than 5 years of working experience with workplace inside the laboratories.

### The reliability and validity of OCAI

Table 1 indicates that all correlations are consistent with the CVF and statistically significant in both current and expected culture. For instance, in the current culture, the correlation between the clan and adhocracy culture is − 0.44, clan and market culture is − 0.39, clan and hierarchy culture is − 0.43, adhocracy and market culture is − 0.10, adhocracy and hierarchy culture is − 0.12, market and hierarchy culture is − 0.29. In the expected OC, the correlations between dimensions are also significant, but most of the correlations are negative. The data is therefore appropriate for factor analysis.

**Table 1:** Correlations of the OC Dimensions

Culture	Current OC				Expected OC			
	1	2	3	4	1	2	3	4
1. Clan Culture	–				–			
2. Adhocracy Culture	−0.44*	–			−0.30*	–		
3. Market Culture	−0.39*	−0.10*	–		−0.60*	0.18*	–	
4. Hierarchy Culture	−0.43*	−0.12*	− 0.29*	–	−0.18*	− 0.50*	−0.23*	–

*p < 0.05

Means, standard deviations and Cronbach’s alphas for each of the current culture factors and expected culture factors are shown in Table 2. In general, clan culture and market culture are favourably chosen by the participants for the present culture (Mean = 26.60). At the same time, participants chose clan culture as the expected culture for future workplace (Mean = 31.50). Each Cronbach’s alphas coefficient is satisfactory compared to normal standards of reliability, statistical significantly. In current culture, the coefficient is 0.80 for the clan culture and 0.60 for the remaining. In expected culture, the coefficient for the clan, the adhocracy, the hierarchy, and the market culture is 0.70, 0.70, 0.70 and 0.60, respectively. In other terms, respondents are more likely to rate their organisation’s culture consistently across the various questions on the instrument.

**Table 2:** Means and Standard Deviations of Current Culture Factors and Expected Culture Factors

Culture types	Mean		SD		Minimum		Maximum		α Reliability	
	CC	EC	CC	EC	CC	EC	CC	EC	CC	EC
Clan Culture	26.60	31.50	8.08	7.80	9.20	17.50	68.30	65.00	0.80	0.70
Adhocracy Culture	24.02	23.80	5.50	6.10	5.00	0	40.00	55.00	0.60	0.70
Hierarchy Culture	22.50	19.90	5.70	6.40	6.70	0	53.20	50.00	0.60	0.70
Market Culture	26.6	24.80	6.20	6.80	5.00	7.50	45.00	61.70	0.60	0.60

*CC* Current Culture, *EC* Expected culture

Table 3 shows the results of CFA analysis for both current and expected culture data to assess the model fit indices. We tested all the prior assumptions and had the met results for conducting the analysis. The unidimensional models showed the χ^2^/*df* for both current and ideal culture data is higher than three as a good model fit required. Therefore, the unidimensional models are not considered a good model fit for both current and ideal data. The CFA for the four-factor CC and EC data seemed to have a marginal good fit (χ^2^/*df* < 3; SRMR varied from ten to 14; CFI was below 0.8, RMSEA = 0.12 for EC).

**Table 3:** Comparisons of Fit Indices between the Unidimensional and Conventional Models of Current and Expected Culture

Fit indices	Df	χ2	p	*SRMR*^a^	CFI^b^	RMSEA^c^
Models						
Current Culture						
Uni^d^	252	7660.16	.000	15.16	0.14	0.23
Four factor	246	649.1	.001	10.0	70.0	.10
∆ Uni^d^ - Four factor model	9	7011.06	.000			
Expected Culture						
Uni^d^	252	7326.35	.000	18.40	.17	.22
Four factor model	246	626.50	.001	14.00	.80	.12
∆ Uni^d^ - Four factor model	6	6699.85	.000			

Note. ^a^Standardised Root Mean Square Residual. ^b^Comparative Fit Index. ^c^Root Mean Square Error of Approximation. ^d^Unidimensional

**Fig. 1:**
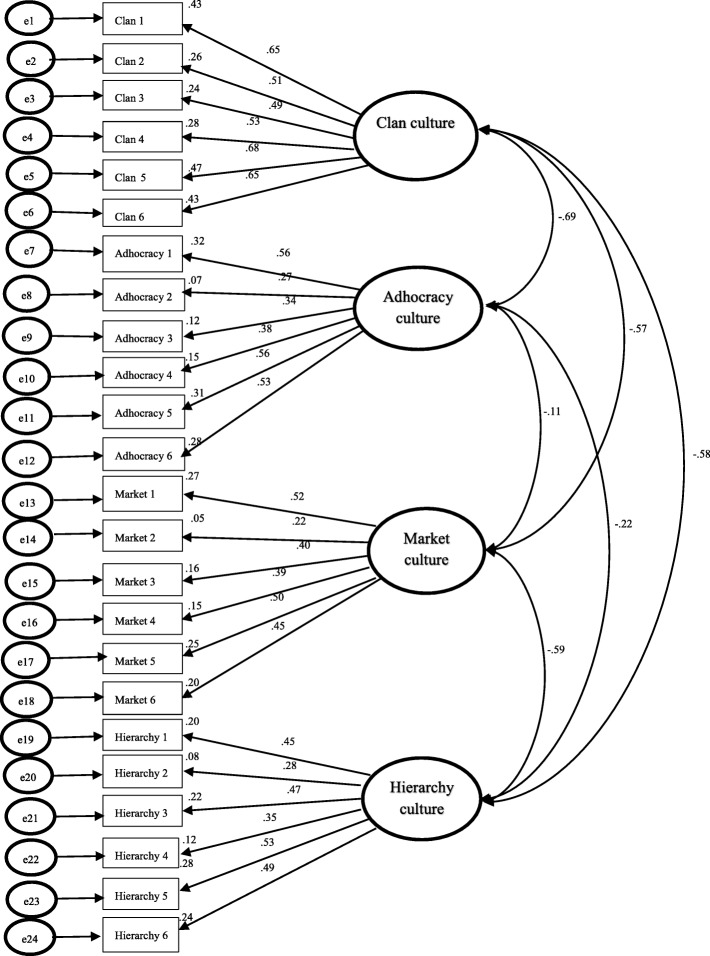
CFA Results for Conventional Four-Factor OCAI Model of Current OC

**Fig. 2:**
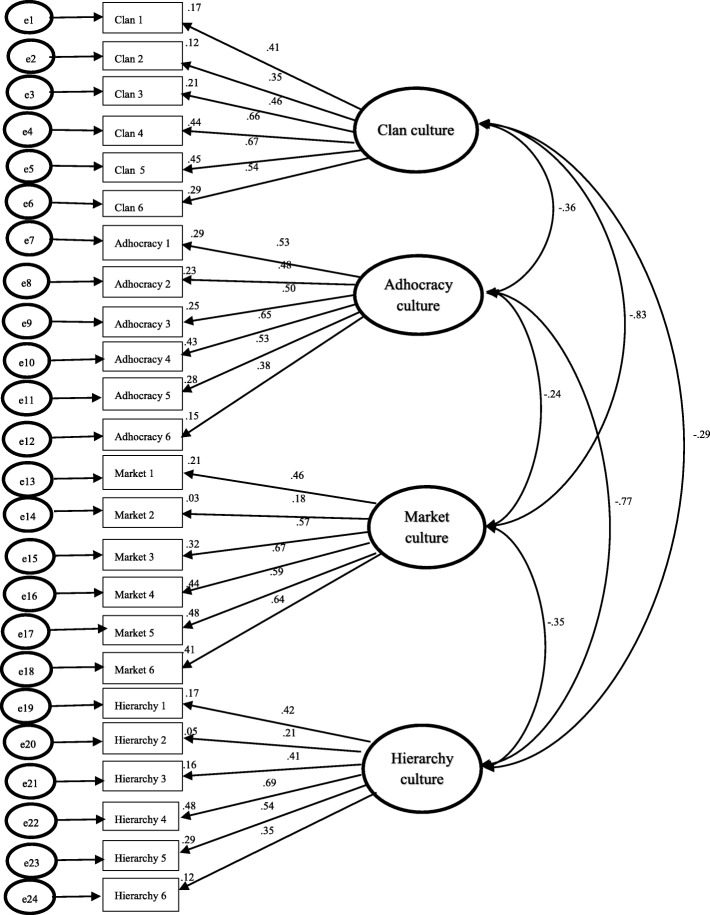
CFA Results for Conventional Four-Factor OCAI Model of Expected OC

Figures 1 and 2 illustrated a model re-assessment for CC and EC model using AMOS version 20.0. As seen in the CC data, the factor loading coefficients are in moderate correlation (0.3 < β < 0.5) except for Adhocracy two, Market two and Hierarchy two (β = 0.27; β = 0.22; β = 0.28); the correlations between factors are negative; most correlations between factors are moderate to strong. Regarding the EC data, the factor loading coefficients of Market two and Hierarchy two are under moderate (β = 0.19; β = 0.21). Most correlations between factors are strong except for the correlations between Clan to Market which are slightly weak. These results suggest the OCAI to be of fair construct validity.

## Discussion

To our knowledge, this study is the first to validate the OCAI in healthcare setting in a developing country as Vietnam. The study suggests that the Clan culture stands out to be the most dominant one in both current and expected culture, followed by the Hierarchy culture. The result shows the internally focus in organization, meaning that the respondents highly prefer the value of internal stability rather than having a position in a competitive context, which is commonly in state organisations or governmental organizations. There was a dearth of research data on OC in Vietnam. We identified only one study on OC which was conducted in a local commercial joint stock bank, Sacombank, in southern Vietnam [17]. Our study is consistent with that study in that Clan culture was most expected, followed by other culture types. However, in terms of current culture, Phuong found that hierarchy culture was reported as most commonly, while our study indicates that Clan culture remains dominant. This difference is understandable given different features between two settings – a for-profit, private economic entity versus a not-for-profit, public healthcare organization. To compare with other countries, our result is comparable with a study which administered the OCAI to 87 nurses [16] and another study with the measurement of OC at the United states [2]. Also, a study conducted in 7 research laboratories affiliated with the University of Indonesia indicated that clan culture was the most common and was desired among member of research laboratory [1]. The CFA results of this study suggest that Clan culture has the highest correlation with the remaining culture, especially with the Market culture even though they lead in two opposite theoretical dimensions. All of the correlations were different from zero, even though there were a few implying weak relationships between these culture type factors. Thus, it could be said that the OC of hospital in Vietnam is the balance of all four types of culture, with the dominant type being Clan culture. Such balance might be due to the natural essence of public hospitals in Vietnam. Clan culture was most preferred because the staff acknowledge the organization as their second family, where they can share the common goals and values with the expectation of being empowered by their leaders. The trusting and dependent relationships between the leaders and members of organisation also boost the adaptability, creativity and innovation of the hospital [20, 21]. Apart from that, the public hospital, which was established and managed by the government, had to set out, to a certain extent, the principles and rules for the members to abide to. Market culture is also a dominant trait. Indeed, the current Government policy has been shifting the hospital financing from being subsidised to self-reliant, which enables the organisation to set their own economic goals.

Prior to our study, the validation of OCAI had been conducted for the English version [9] and the Korean version [5]. Choi and colleagues validated the Korean version of OCAI by employing factor analysis and found moderate model fit with acceptable psychometric properties upon accepting a small sample size bias [5]. Meanwhile, Heritage and colleagues validated the use of OCAI within the Australian context. By using CFA, the study came to a conclusion that good model fit was acknowledged for both current and expected cultures [9]. The findings regarding the Vietnamese version of OCAI that we used in this study show fairly good internal consistency, which is demonstrated by high Cronbach’s alpha coefficients, ranging from 0.6 to 0.8 in both current and expected culture. The results were on par with the previous validation studies of OCAI [5, 9]. Moreover, it also indicates that the four-factor model has a higher fit than the unidimensional model in both current and expected culture. Such view was shared by the validation study in Australia [9]. However, only some of the CFA indexes demonstrated the fairly marginal good fit of the same four-factor model for the application of OCAI in Vietnam health care settings. The results of CFA are also supportive of the fairly good fit of the model as the factor loading coefficients were in moderate correlation (0.3 < β < 0.5) and some are under moderate. The loading factor is lower compared to the study by Heritage and colleagues [9]. There are multiple explanatory causes for the model getting only marginally good fit. It could be due to the translation of OCAI to the Vietnamese version used in this study. It came to our attention that the item two from CFA in both EC and CC had relatively poor fit in comparison with other items (Market two and Hierarchy two in EC and Adhocracy two, Market two and Hierarchy two in CC). Even though the translation was precise enough to carry the same meaning as the English version, we suspect that there would still be dissimilarities between the translated and the original content regarding the meanings and concepts of the words. Indeed, in some cases, several English words have the same meaning as one Vietnamese word or vice versa. Apart from that, the mechanism of wording and sentence structure is also different between the two languages. Misinterpretation of even a single word used in psychological instruments could create biases in the results or a methodological error that could have adverse effects on the findings of the studies [5]. Therefore, the Vietnamese OCAI version used in this study might need further modification in order to have smaller dissimilarities with the English version and become a more precise OC assessment tool in Vietnam.

This study has some limitations that need to be considered when interpreting the results. Although efforts were made to include a relatively large number of respondents, the sample of this study might not be representative for the whole health care settings of the country. In addition, as the study was conducted in a public hospital, the respondents working here might consider their organisation to be more of a Hierarchy culture. Future research might need to take on a cross-national sample with respondents working not only in the public but also the private sector in order to have a better representation of the health care settings in Vietnam.

## Conclusions

The Competing Values Framework (CVF), which originally proposed [19] and has advanced the measurement and comprehension of OC structure. Based on it, the OC Assessment Instruments (OCAI) was developed to measure the OC aspects in the situation of present and employee’s wishes. Up to date, this instrument has been applied in different contexts around the world but in Vietnam, there is only one study applying this instrument to assess the OC in the banking sector. Our study aimed to validate the OCAI in healthcare setting of a developing country like Vietnam as a first step toward establishing a valid Vietnamese version of the OCAI and a valid basic for future studies in the field of measuring and managing OC. The results indicated that the OCAI had quite good reliability with Cronbach’s alpha coefficients ranging from 0.6 to 0.8 in both current and expected culture. Regarding its validity, the translated version of OCAI showed a fairly good fit, with most of CFA loading factors of less than 0.60. These findings suggest that OCAI could be used to measure the OC. Future studies may also need to validate the OCAI in different sectors and settings.

## Data Availability

The datasets generated during and/or analysed during the current study are available from the corresponding author on the reasonable request.

## Abbreviations

CC: Current Culture
CFA: Confirmatory Factor Analysis
CFI: Comparative fit index
CVF: Competing Values Framework
EC: Expected Culture
OC: Organizational Culture
OCAI: Organizational Culture Assessment Instrument
RMSEA: Root mean square error of approximation
